# Supplementary material for: Linguistic Analysis for Identifying Depression and Subsequent Suicidal Ideation on Weibo: Machine Learning Approaches
Source: Int J Environ Res Public Health. 2023 Feb 2;20(3):2688. doi: 10.3390/ijerph20032688 (PMC9915029; doi:10.3390/ijerph20032688)
Supplement: Supplementary file 1 [file ijerph-20-02688-s001.zip › ijerph-2110889-supplementary.pdf]

Table S1. The full logistic regression model of using the simplified Chinese version of LIWC (SCLIWC) features to classify the depression and control groups.

SCLIWC features	*β*	*S.E.*	*Z*	*p*	*sig*
(Intercept)	-0.47	0.06	-8.00	0.00	***
I	1.02	0.10	10.52	< 2e-16	***
We	0.14	0.06	2.14	0.03	*
You	0.01	0.07	0.21	0.83	
SheHe	-0.09	0.06	-1.48	0.14	
They	-0.01	0.06	-0.20	0.84	
iPron	0.09	0.07	1.19	0.23	
Verb	0.10	0.12	0.86	0.39	
AuxVerb	-0.09	0.13	-0.65	0.52	
Adverb	0.02	0.13	0.18	0.86	
Preps	0.07	0.07	1.01	0.31	
Conj	0.03	0.09	0.35	0.73	
Negate	-0.06	0.06	-1.04	0.30	
Quant	-0.20	0.06	-3.59	0.00	***
Number	0.10	0.05	1.85	0.06	
Swear	-0.05	0.07	-0.76	0.45	
YouPL	-0.02	0.06	-0.36	0.72	
PrepEnd	0.21	0.06	3.48	0.00	***
SpecArt	-0.30	0.06	-5.09	0.00	***
QuanUnit	-0.04	0.06	-0.70	0.49	
Interjunction	0.01	0.11	0.07	0.95	
MultiFun	-0.26	0.09	-2.96	0.00	**
PastM	-0.13	0.07	-1.84	0.07	
PresentM	0.11	0.07	1.67	0.09	
FutureM	0.02	0.06	0.40	0.69	
ProgM	-0.14	0.09	-1.55	0.12	
Social	0.23	0.08	2.80	0.01	**
Family	-0.16	0.06	-2.70	0.01	**
Friend	0.07	0.05	1.43	0.15	
Humans	-0.06	0.06	-1.07	0.29	
Affect	0.40	0.13	3.00	0.00	**
PosEmo	-0.25	0.10	-2.43	0.02	*
NegEmo	0.40	0.12	3.23	0.00	**
Anx	0.15	0.08	1.87	0.06	
Anger	-0.12	0.08	-1.65	0.10	
Sad	0.41	0.09	4.44	0.00	***
Insight	0.06	0.07	0.90	0.37	
Cause	0.07	0.07	1.11	0.27	
Discrep	0.51	0.13	4.04	0.00	***
Tentat	0.34	0.10	3.41	0.00	***
Certain	0.05	0.07	0.78	0.44	

Inhibition	-0.09	0.06	-1.47	0.14	
Inclusive	0.02	0.07	0.24	0.81	
Exclusive	-0.35	0.10	-3.57	0.00	***
Percept	-0.40	0.11	-3.71	0.00	***
See	0.38	0.11	3.48	0.00	***
Hear	0.17	0.07	2.34	0.02	*
Feel	-0.06	0.06	-0.91	0.36	
Bio	-0.06	0.10	-0.57	0.57	
Body	0.05	0.08	0.69	0.49	
Health	0.70	0.09	7.95	0.00	***
Sexual	-0.11	0.06	-1.91	0.06	
Ingest	-0.26	0.09	-2.88	0.00	**
Relative	-0.40	0.11	-3.80	0.00	***
Motion	0.32	0.07	4.89	0.00	***
Space	0.10	0.09	1.11	0.27	
Time	-0.15	0.08	-1.86	0.06	
Work	-0.19	0.06	-2.92	0.00	**
Achieve	-0.30	0.07	-4.33	0.00	***
Leisure	-0.01	0.06	-0.15	0.88	
Home	0.00	0.06	0.01	0.99	
Money	-0.21	0.08	-2.67	0.01	**
Religion	-0.09	0.06	-1.41	0.16	
Death	0.36	0.08	4.37	0.00	***
Assent	-0.05	0.10	-0.55	0.59	
Nonfl	0.21	0.08	2.60	0.01	**
Filler	-0.28	0.08	-3.57	0.00	***
Psychology	-0.01	0.06	-0.25	0.80	
Love	-0.11	0.06	-1.82	0.07	
tPast	0.07	0.07	1.00	0.32	
tNow	-0.04	0.07	-0.66	0.51	
tFuture	0.10	0.08	1.30	0.19	
Period	-0.15	0.07	-2.15	0.03	*
Comma	-0.28	0.06	-4.68	0.00	***
Colon	-0.10	0.06	-1.63	0.10	
SemiC	-0.29	0.08	-3.51	0.00	***
QMark	-0.09	0.08	-1.11	0.27	
Exclam	-0.22	0.07	-3.29	0.00	**
Dash	0.31	0.08	3.77	0.00	***
Quote	-0.27	0.08	-3.25	0.00	**
Apostrophe	-0.11	0.11	-0.99	0.32	
Parenth	0.18	0.07	2.69	0.01	**
OtherP	-0.17	0.07	-2.38	0.02	*
WordCount	1.00	0.07	14.54	< 2e-16	***
WordPerSentence	-0.59	0.20	-3.02	0.00	**

RateDicCover	-0.30	0.11	-2.82	0.00	**
RateNumeral	-0.70	0.09	-8.11	0.00	***
RateSixLtrWord	-0.11	0.09	-1.27	0.20	
RateFourCharWord	-0.19	0.08	-2.31	0.02	*
RateLatinWord	0.38	0.08	4.52	0.00	***
NumEmotion	-0.76	0.10	-7.61	0.00	***
NumHashTag	0.42	0.08	5.42	0.00	***
NumURLs	0.29	0.07	4.24	0.00	***

****** $p < 0.001$, ** $p < 0.01$, * $p < 0.05$***
